# Immunocheckpoint Inhibitor- (Nivolumab-) Associated Hypereosinophilia in Non-Small-Cell Lung Carcinoma

**DOI:** 10.1155/2020/7492634

**Published:** 2020-05-15

**Authors:** Navdeep Singh, Sandeep Singh Lubana, George Constantinou, Andrea N. Leaf

**Affiliations:** ^1^Medicine/Division of Hospice/Palliative Care, North Shore University Hospital, 300 Community Dr., Manhasset, NY 11030, USA; ^2^Medicine/Division of Hematology/Oncology, SUNY Downstate Medical School, 450 Clarkson Ave., Brooklyn, NY 11203, USA; ^3^Lafayette General Medical Center, 1214 Coolidge St., Lafayette, LA 70503, USA; ^4^Medicine/Division of Hematology/Oncology, Brooklyn VA Medical Center, 800 Poly Pl., Brooklyn, NY 11209, USA

## Abstract

Immunocheckpoint inhibitor (ICI) therapy has provided significant clinical improvements in the treatment of several malignancies. The purpose of this report is to increase awareness of hypereosinophilia associated with checkpoint inhibitors, a topic that has been rarely reported. Hypereosinophilia may need to be addressed especially if eosinophil counts increase to levels where hypereosinophilic visceral complications can occur. We are presenting a case of a 57-year-old male with hypereosinophilia that was seen in the setting of progression of metastatic non-small-cell lung cancer during and after nivolumab treatment.

## 1. Introduction

The use of immunotherapeutic agents has proven to be effective for patients with many different types of cancers [1–3]. The antitumor function of T-cells is inhibited by PD-L1 which is expressed on many malignant tumors. Nivolumab, a fully human IgG4 monoclonal antibody against PD-1 receptors, blocks the interaction of PD-1 on the T-cell and PD-L1/PD-L2 on the tumor cell improving the antitumor function of the T-cells. The US FDA has approved nivolumab for the treatment of several malignancies including non-small-cell lung cancer [1, 2, 4, 5].

Known toxicities for checkpoint inhibitors are typically immune-mediated, and guidelines have been published for the management of the immune-related adverse event (irAE) [6]. irAEs are well known with nivolumab as well as other ICIs. Eosinophilia has also been reported with the use of ICIs. [7] Although PD-L1 is widely used as a biomarker to predict the response to ICIs, responses have been reported in patients having tumors without any PD-L1 expression [8].

Eosinophilia in patients with melanoma has been reported as a biomarker for tumor response to ICIs [9, 10]. The partial response of the tumor in metastatic disease has been postulated to be secondary to eosinophilia as a result of immunotherapy [11]. Eosinophilia in patients with lung cancer who received immunotherapy have been reported to have had partial response to nivolumab [7]. Herein, we report a case of hypereosinophilia with nivolumab therapy in a patient with progression of metastatic NSCLC. The role of eosinophilia as a biomarker requires additional investigation.

## 2. Case Presentation

The patient is a 57-year-old male with an extensive smoking history who underwent right upper lobe lobectomy in May 2012 for a clinical stage I adenocarcinoma of the lung. He was found to have microscopic ipsilateral mediastinal adenopathy. He received adjuvant chemotherapy with pemetrexed and cisplatin followed by radiation therapy for his pathologic stage IIIA (pT2aN2M0) adenocarcinoma of the lung. A positron emission tomography (PET) scan in February 2013 did not show any evidence of malignancy.

One year after completion of adjuvant chemotherapy, in October 2013, the patient developed headaches. Magnetic resonance imaging (MRI) of the brain was consistent with four intracranial metastases. PET/CT scan revealed several subcentimeter metastatic pulmonary nodules. EGFR/ALK/ROS1 testing at that time did not reveal any targetable mutations. He underwent whole brain radiation therapy.

**Figure 1:**
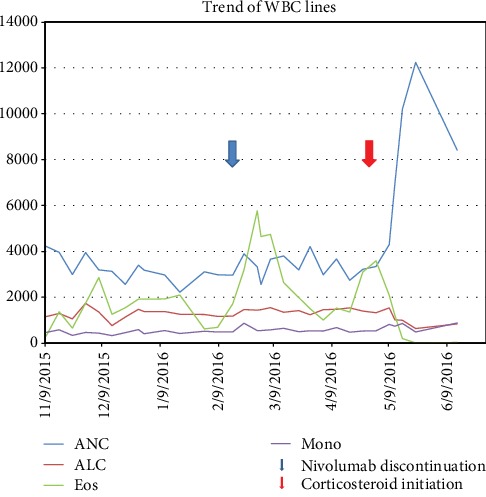
Graph depicting various white cell lines following initiation of immunotherapy (nivolumab) in November 2015.

In the next two years, the patient had progression of disease (POD) in the lung through several lines of chemotherapy. He also developed CNS progression with three new lesions in December 2014, for which he underwent stereotactic radiation therapy (SRS). Six months later, the patient developed two more intracranial lesions for which he again received SRS. New intracranial subcentimeter metastatic disease was identified in September 2015 which was not amenable to further radiation. Immunotherapy with the checkpoint inhibitor nivolumab was initiated in November 2015 (Figure 1 and Table 1).

Eosinophil counts dating back to 1998 had always been within normal limits except for a brief period of mild increased eosinophilia after adjuvant chemotherapy in 2012 which spontaneously resolved. Four weeks after initiation of nivolumab, his absolute eosinophil count was noted to be elevated at 2.86 × 10^9^/L; all other hematopoietic cell lines remained unaffected. He had denied any travel within the previous five years and denied any exposure to any known allergens, new products, or new medications. He was asymptomatic, and on physical examination, there was no evidence of skin rash or splenomegaly.

On a follow-up visit in April 2016, after eight cycles of nivolumab, the peripheral blood smear revealed markedly increased eosinophils, but no other significant findings.

Further work-up of the eosinophilia was performed and was unrevealing. Multiple stool samples were obtained, and testing for culture, ova, and parasites remained negative on three separate occasions as was testing for Clostridium difficile toxin. Serum IgG for Strongyloides and serum QuantiFERON test were negative. Liver function tests, thyroid function tests, cortisol, and B12 levels were also normal. Echocardiogram, cardiac enzymes, and EKG did not reveal any abnormalities. Evaluation with bone marrow biopsy or molecular/cytogenetic testing was not pursued as it was felt that there was a clear temporal association of eosinophilia with nivolumab administration. MRI of the brain in February 2016 revealed continued mild progression of metastatic lesions and a new lesion in the left temporal lobe. The PET/CT at that time was consistent with systemic progression in the bone and lung. Given the degree of eosinophilia and evidence of progression, the decision was made to hold further nivolumab therapy in March of 2016.

As the eosinophilia (3.5 × 10^9^/L) was significant and persisted despite discontinuation of nivolumab, the patient was started on prednisone 10 mg daily in May 2016. Repeat MRI of the brain revealed an increase in the size of known intracranial metastases within the infratentorial and supratentorial regions. There was also a new area of edema and gyral-based enhancement with associated mass effect within the superior posterior left temporal lobe; this pattern of enhancement was considered atypical for metastatic disease. The differential included radiation related necrosis, encephalitis/cerebritis, or postinflammatory process.

Given the concern for possible immune-mediated encephalitis/cerebritis, the patient was started on dexamethasone 4 mg twice a day at that time which was later increased to 4 mg three times a day, with a resolution of eosinophilia in two weeks. Two weeks later, in June 2016, the patient started developing thrombocytopenia. Evaluation of the peripheral smear confirmed thrombocytopenia with myelocytes, metamyelocytes, few nucleated red cells, and teardrop cells which was felt to be consistent with a possible myelophthisic process from marrow infiltration of the tumor. A repeat MRI of the brain two weeks later revealed a decrease in edema, the mixed response of lesions with some increasing and some decreasing in size, and the greater confluence of the left temporal lobe lesion which however remained stable in size.

The next day, the patient presented to the emergency room with worsening shortness of breath. The computerized tomography angiogram of the chest was consistent with marked progression of the disease. Despite treatment with broad-spectrum antibiotics and aggressive treatment in the intensive care unit, the patient expired one week after his admission with the progression of the disease. An autopsy not performed as per the family's request.

## 3. Discussion

Eosinophilia with PD-1/PD-L1 checkpoint inhibitors is a rarely reported adverse event. Absolute eosinophilic count (AEC) of more 500 cells/*μ*L in the peripheral blood is eosinophilia, and based on the eosinophil count, it is further subdivided as mild 0.5‐1.5 × 10^9^/L, moderate 1.5‐5 × 10^9^/L, and severe >5 × 10^9^/L [12]. AEC > 1500 × 10^9^/L in the peripheral blood on two separate occasions at least one month apart is defined as hypereosinophilia. Pathologic confirmation of tissue hypereosinophilia is also termed as hypereosinophilia. The term hypereosinophilic syndrome is used when eosinophilia is associated with tissue and organ damage [13]. Early identification of drug-induced hypereosinophilia is critical, especially when deciding whether to continue the drug and/or to treat with corticosteroids.

Allergic or immunologic processes like asthma, eosinophilic granulomatosis with polyangiitis, bronchopulmonary aspergillosis, and helminthic parasitic infections are associated with hypereosinophilia. Hematologic or neoplastic disorders (adenocarcinomas, Hodgkin lymphoma, and T-cell lymphoma) can also lead to hypereosinophilia, but are an uncommon cause [12].

However, hypereosinophilia associated with immune-checkpoint inhibitors has rarely been reported. To date, only five reports of nivolumab-induced hypereosinophilia have been reported in the literature [7, 11, 14, 15].

The prognostic significance of hypereosinophilia associated with nivolumab in terms of overall survival and progression-free survival remains largely undefined. There are reports of advanced lung adenocarcinoma with favorable prognosis when eosinophilia was reported with nivolumab use [7, 14].

In some case reports, eosinophilia was noted as an adverse event and as a prodrome in patients who were later diagnosed with checkpoint-mediated immune complications such as adrenal insufficiency [11] systemic symptom syndrome and hypophysitis [16].

Various studies related to tumor-associated tissue eosinophilia (TATE) in many solid tumors (colorectal and esophageal squamous cell carcinoma) have revealed it to have good prognostic value, but in Hodgkin lymphoma, it is associated with poor prognosis [17–19]. There has been some conflicting data [20] regarding oral SCC and cervical carcinoma where TATE has been shown to be associated with poor prognosis. The mechanism that can explain these effects remains unclear. Tumoricidal properties of eosinophils remain unknown; however, one suggested possible mechanism is the direct cytotoxic effect from degranulation of eosinophilic granules [20].

The role of eosinophils in antitumor immune response has been suggested. The various suggested mechanisms are direct antitumor effects, dendritic cell activation and recruitment, T-cell recruitment and polarization using chemokines and enhanced immune surveillance, and normalization of the tumor microenvironment vasculature [21–24]. Eosinophilia which has been reported in acute or chronic graft-versus-host disease further points towards its immune-related properties [20, 25].

A retrospective study reported a correlation of enhanced immune response, prolonged prostate cancer-specific survival, and trend towards improved overall survival in patients with eosinophilia. Eosinophilia was reported in 28% of patients (105 of 377) following sipuleucel-T treatment at week 6 with resolution by week 14 [26].

A prospective study of 73 patients with advanced melanoma treated with ipilimumab (anticytotoxic T lymphocyte-associated antigen [CTLA] 4 monoclonal antibody) demonstrated correlation of improved overall survival with an increase in eosinophil count of more than 100 cells/*μ*L [10]. Studies of anti-PD-1 monoclonal antibody (pembrolizumab and nivolumab) in patients with melanoma demonstrated a positive correlation between an elevated eosinophil count and overall survival [27, 28]. The median eosinophil count peak (approximately 1000 cells/*μ*L) has been correlated with an improved overall response rate [29]. There are studies contradicting the beneficial effect of eosinophilia. A study with 156 patients with advanced melanoma treated with ipilimumab at a dose of 3 mg/kg manifested immune-related adverse events including eosinophilia without overall survival improvement [30]. T-cell-mediated antitumor response could be affected by activated eosinophils via enhancing CD8-T-cell infiltration as demonstrated in a mouse model [23]. Therefore, some experts recommend that eosinophilia should not be used as a prognostic factor due to its codependence on T-cells.

Alternatively, eosinophilia could simply be due to an allergic drug reaction. There has been a reported case of drug-related eosinophilia and systemic symptom (DRESS) syndrome in a patient with melanoma following ipilimumab and nivolumab administration [15].

To determine the significance of eosinophils in immunotherapy requires additional studies. To our knowledge, there are only two reports of hypereosinophilia associated with nivolumab used in non-small-cell lung cancer where patients had a favorable response [7, 14]. However, in our case, the hypereosinophilia associated with nivolumab use is in the treatment of lung adenocarcinoma resulted in progression of the disease.

In the case described here, the patient remained asymptomatic despite a high eosinophil count (3.6 × 10^9^/L), and no effects were noted on other leukocyte lineages. The significant and persistent hypereosinophilia was felt to be temporally related to the administration of nivolumab. It is unclear whether the MRI changes in the brain were secondary to immune cerebritis as there was also clear progression of his metastatic disease. Given the progression of the disease and the potential toxicity of the drug, nivolumab was discontinued. The more challenging question is whether it is warranted to discontinue nivolumab in the setting of very severe hypereosinophilia when a clinical response is being observed [14]. Notably, in this case after nivolumab was held, the eosinophilia did not resolve. As such, we decided to treat the patient with corticosteroids. Hypereosinophilia with nivolumab may be more common than reported, as it can potentially be overlooked in an asymptomatic patient. It is a complication, however, that may need to be addressed especially if eosinophil counts increase to levels where hypereosinophilic visceral immune-related complications occur. As it may be an early marker for later potential immune-related complications, it would also allow clinicians to have increased vigilance for those patients.

## 4. Conclusion

The prognostic significance of hypereosinophilia associated with nivolumab in terms of overall survival and progression-free survival remains largely undefined. This is the first case of a non-small-cell lung cancer (NSCLC) patient in which hypereosinophilia was associated with unfavorable tumor response to an immune checkpoint inhibitor. Further investigations in a larger patient population is warranted to demonstrate eosinophilia as a prognostic biomarker of immunotherapy.

## Figures and Tables

**Table 1:** 

Timeline:	ANC	ALC	Eos	Mono	Hg	PLT
11/09/2015	4220	1160	300	470	10.9	189
11/16/2015	3960	1300	1370	580	11.4	163
11/23/2015	2990	1060	660	340	12	174
11/30/2015	3950	1740	1740	470	12.7	194
12/07/2015	3190	1360	2860	440	12.1	196
12/14/2015	3130	770	1260	330	10.8	169
12/21/2015	2560	1140	1540	460	12.5	157
12/28/2015	3400	1480	1920	590	13.5	204
12/31/2015	3180	1380	1920	410	13.1	198
1/11/2016	2970	1380	1930	550	12.7	154
1/19/2016	2220	1260	2100	420	12.1	147
2/01/2016	3110	1250	620	520	13.5	189
2/08/2016	2980	1170	690	490	13.1	163
2/16/2016	2960	1180	1710	490	13.6	164
2/22/2016	3890	1470	3200	870	14.4	186
2/29/2016	3330	1440	5770	550	13.1	181
3/02/2016	2560	1460	4650	550	13.1	194
3/07/2016	3660	1550	4740	580	12.5	168
3/14/2016	3800	1350	2640	650	13.4	187
3/22/2016	3190	1420	1990	500	13.4	217
3/28/2016	4210	1240	1500	540	12.8	171
4/04/2016	2980	1460	1010	530	13	159
4/11/2016	3670	1480	1530	680	12.9	188
4/18/2016	2740	1540	1360	480	12.5	170
4/25/2016	3220	1400	3100	530	12.3	179
5/02/2016	3340	1330	3590	540	12.9	186
5/09/2016	4300	1540	2090	820	12.9	205
5/12/2016	6950	1020	1210	740	12.8	204
5/16/2016	10210	1000	200	860	13.2	198
5/23/2016	12240	640	10	490	12.8	175
6/14/2016	8420	840	30	880	13.5	106
6/29/2016	6230	620	20	200	13.3	111

Abbreviations: ANC: absolute neutrophil count; ALC: absolute lymphocyte count; Eos: eosinophil count; Mono: monocyte count; Hg: hemoglobin in mg/dL; PLT: platelet count.

## References

[1] Hodi F. S., O'Day S. J., McDermott D. F. (2010). Improved survival with ipilimumab in patients with metastatic melanoma. *The New England Journal of Medicine*.

[2] Topalian S. L., Hodi F. S., Brahmer J. R. (2012). Safety, activity, and immune correlates of anti-PD-1 antibody in cancer. *The New England Journal of Medicine*.

[3] Larkin J., Chiarion-Sileni V., Gonzalez R. (2015). Combined nivolumab and ipilimumab or monotherapy in untreated melanoma. *The New England Journal of Medicine*.

[4] Chen R., Zinzani P. L., Fanale M. A. (2017). Phase II study of the efficacy and safety of pembrolizumab for relapsed/refractory classic Hodgkin lymphoma. *Journal of Clinical Oncology*.

[5] Hammers H. J., Plimack E. R., Infante J. R. (2017). Safety and efficacy of nivolumab in combination with ipilimumab in metastatic renal cell carcinoma: the CheckMate 016 study. *Journal of Clinical Oncology*.

[6] Brahmer J. R., Lacchetti C., Thompson J. A. (2018). Management of immune-related adverse events in patients treated with immune checkpoint inhibitor therapy: American Society of Clinical Oncology clinical practice guideline Summary. *Journal of Oncology Practice*.

[7] Lou Y., Marin-Acevedo J. A., Vishnu P. (2019). Hypereosinophilia in a patient with metastatic non-small-cell lung cancer treated with antiprogrammed cell death 1 (anti-PD-1) therapy. *Immunotherapy*.

[8] Meng X. J., Huang Z. Q., Teng F. F., Xing L. G., Yu J. M. (2015). Predictive biomarkers in PD-1/PD-L1 checkpoint blockade immunotherapy. *Cancer Treatment Reviews*.

[9] Hopkins A. M., Rowland A., Kichenadasse G. (2017). Predicting response and toxicity to immune checkpoint inhibitors using routinely available blood and clinical markers. *British Journal of Cancer*.

[10] Delyon J., Mateus C., Lefeuvre D. (2013). Experience in daily practice with ipilimumab for the treatment of patients with metastatic melanoma: an early increase in lymphocyte and eosinophil counts is associated with improved survival. *Annals of Oncology*.

[11] Ariyasu R., Horiike A., Yoshizawa T. (2017). Adrenal insufficiency related to anti-programmed death-1 therapy. *Anticancer Research*.

[12] Roufosse F., Weller P. F. (2010). Practical approach to the patient with hypereosinophilia. *The Journal of Allergy and Clinical Immunology*.

[13] Valent P., Klion A. D., Horny H. P. (2012). Contemporary consensus proposal on criteria and classification of eosinophilic disorders and related syndromes. *Journal of Allergy and Clinical Immunology*.

[14] Osawa H., Okauchi S., Taguchi S., Kagohasi K., Satoh H. (2018). Immuno-checkpoint inhibitor associated hyper-eosinophilia and tumor shrinkage. *Tuberkuloz ve Toraks*.

[15] Mirza S., Hill E., Ludlow S. P., Nanjappa S. (2017). Checkpoint inhibitor associated drug reaction with eosinophilia and systemic symptom syndrome. *Melanoma Research*.

[16] Okano Y., Satoh T., Horiguchi K. (2016). Nivolumab-induced hypophysitis in a patient with advanced malignant melanoma. *Endocrine Journal*.

[17] Fernandez-Acenero M. J., Galindo-Gallego M., Sanz J., Aljama A. (2000). Prognostic influence of tumor-associated eosinophilic infiltrate in colorectal carcinoma. *Cancer*.

[18] Ishibashi S., Ohashi Y., Suzuki T. (2006). Tumor-associated tissue eosinophilia in human esophageal squamous cell carcinoma. *Anticancer Research*.

[19] von Wasielewski R., Seth S., Franklin J. (2000). Tissue eosinophilia correlates strongly with poor prognosis in nodular sclerosing Hodgkin’s disease, allowing for known prognostic factors. *Blood*.

[20] Davis B. P., Rothenberg M. E. (2014). Eosinophils and cancer. *Cancer Immunology Research*.

[21] Jacobsen E. A., Helmers R. A., Lee J. J., Lee N. A. (2012). The expanding role(s) of eosinophils in health and disease. *Blood*.

[22] Simson L., Ellyard J. I., Dent L. A. (2007). Regulation of carcinogenesis by IL-5 and CCL11: a potential role for eosinophils in tumor immune surveillance. *Journal of Immunology*.

[23] Carretero R., Sektioglu I. M., Garbi N., Salgado O. C., Beckhove P., Hammerling G. J. (2015). Eosinophils orchestrate cancer rejection by normalizing tumor vessels and enhancing infiltration of CD8^+^ T cells. *Nature Immunology*.

[24] Gatault S., Legrand F., Delbeke M., Loiseau S., Capron M. (2012). Involvement of eosinophils in the anti-tumor response. *Cancer Immunology, Immunotherapy*.

[25] McNeel D., Rubio M. T., Damaj G. (2002). Hypereosinophilia as a presenting sign of acute graft-versus-host disease after allogeneic bone marrow transplantation. *Transplantation*.

[26] McNeel D. G., Gardner T. A., Higano C. S. (2014). A transient increase in eosinophils is associated with prolonged survival in men with metastatic castration-resistant prostate cancer who receive sipuleucel-T. *Cancer Immunology Research*.

[27] Moreira A., Leisgang W., Schuler G., Heinzerling L. (2017). Eosinophilic count as a biomarker for prognosis of melanoma patients and its importance in the response to immunotherapy. *Immunotherapy*.

[28] Weide B., Martens A., Hassel J. C. (2016). Baseline biomarkers for outcome of melanoma patients treated with pembrolizumab. *Clinical Cancer Research*.

[29] Bernard-Tessier A., Jeanville P., Champiat S. (2017). Immune-related eosinophilia induced by anti-programmed death 1 or death-ligand 1 antibodies. *European Journal of Cancer*.

[30] Schindler K., Harmankaya K., Kuk D. (2014). Correlation of absolute and relative eosinophil counts with immune-related adverse events in melanoma patients treated with ipilimumab. *Journal of Clinical Oncology*.

